# Inequality of Low Air Quality-Related Health Impacts among Socioeconomic Groups in the World of Work

**DOI:** 10.3390/ijerph191912980

**Published:** 2022-10-10

**Authors:** Thi Phuoc Lai Nguyen, Salvatore G. P. Virdis, Ekbordin Winjikul

**Affiliations:** 1 Department of Development and Sustainability, School of Environment, Resources and Development, Asian Institute of Technology (AIT), P.O. Box 4, Klong Luang, Pathum Thani 12120, Thailand; 2 Department of Information and Communication Technologies, School of Engineering and Technology, Asian Institute of Technology (AIT), P.O. Box 4, Klong Luang, Pathum Thani 12120, Thailand; 3 Department of Energy, Environment and Climate Changes, School of Environment, Resources and Development, Asian Institute of Technology (AIT), Pathum Thani 12120, Thailand

**Keywords:** air pollution perceptions, health symptoms, informal sector workers, Bangkok

## Abstract

This research aimed to assess the perceptions of air quality and health symptoms caused by low urban air quality among vulnerable socio-economic groups in the world of work in Bangkok, Thailand through a questionnaire survey of 400 workers of both formal and informal sectors in the five districts with different socio-economic characteristics and levels of air pollution. The findings showed symmetry between air quality-monitoring data and health symptoms of different socio-economic groups but asymmetry between air quality-monitoring data and people’s perceptions of air quality in their areas. It also showed inequalities of low air quality-related health impacts on socio-economic groups in the world of work. People working near the streets, highways, and industrial zones tended to have more health symptoms related to low air quality, and informal sector workers faced more health risks than formal sector workers. The study appeals for effective air pollution communication to enhance the public and informal sector worker population’s literacy of air pollution, the sources of air pollution and its critical health impacts, and the available and sufficient primary care organizations and community health care centers to address work-related health needs to reach the informal sector worker population.

## 1. Introduction

The Asia-Pacific region experienced the world’s sharpest rise in premature deaths between 1990 and 2015 caused by ambient air pollution (i.e., fine particulate matter in the air) [1]. Air pollution is caused by several sources, including vehicle emissions in cities, biomass burning in rural areas, transboundary haze in border areas, and industrial discharges in concentrated industrialized zones. Air pollution, especially smoke pollution, contains particulate matter with a diameter equal to or less than 2.5 microns (PM_2.5_) that is transmitted into the lungs, causing respiratory problems and other health problems over the long term [2]. Exposure to air pollution over the long term does not only cause physical health problems but also induces lifelong socio-economic handicaps and mental health problems [3,4]. An association between air pollution and mortality has been found in many countries [5,6].

Thailand is one of the top air-polluted countries in the region. The most recent data indicates that the 2019 country’s annual mean concentration of PM_2.5_ is 10–41 µg/m^3^ [7], exceeding the recommended maximum of 10 µg/m^3^ considered unsafe by the World Health Organization (WHO). Moreover, the days that the PM_2.5_ concentration in the Bangkok metropolitan region exceeded the 24-h Thai National Ambient Air Quality Standards from November to April 2019 were higher than those in the previous year’s [7]. Poor air quality has extremely serious health consequences, including cancer, stroke, asthma, or heart disease. 

Thailand has the third highest socio-economic inequality in the world [8]. Thailand has a large informal economic sector which accounts for 42 percent in urban areas and 56 percent of all employment in Thailand [9]. Informal sector workers are vulnerable socio-economic groups [10], often including migrants, poor women, and youths involved in work that is dirty, dangerous, difficult, and low-paid [11]. Informal sector workers are often not covered by social welfare and insurance, such as healthcare, unemployment, disability, and retirement, and have no formal agreements and safety protection at work, no contributions to pensions and medical aid, or the right to paid sick leave or annual leave [9].

There has been increasing policy and scientific attention to inequalities in environmental hazards, including air pollution [12,13]. Inequalities in air pollution exposures have important health implications. UNESCAP confirmed that poor and disadvantaged people are more exposed and vulnerable to the pernicious impact of pollution. Because they are less able to protect themselves from pollution, their health and productivity suffer disproportionately. However, very few studies have been conducted to investigate inequalities in air pollution exposures, including the origins of environmental inequality, and the disproportionately impacted populations of low socioeconomic status and vulnerable people, such as migrants, the elderly, poor women, and youth, and to propose policy implications for environmental justice of air pollution [14]. Socio-economic characteristics of a socio-economic group influence its population’s health and well-being through the availability of public goods and services and environmental pollutants and social protection to cope with health problems [15]. Vulnerable socioeconomic groups are disproportionately more affected by air pollution than other groups. This evidence has been reported by some studies in developed countries [16], but it remains understudied in Southeast Asia and Thailand.

This research aimed to assess the perceptions of air quality and health symptoms caused by urban low air quality among vulnerable socio-economic groups working and living in Bangkok, Thailand. The goal of this research is to inform policymakers, relevant stakeholders, and the public about existing inequalities in low air quality exposure and its impacts among socio-economic groups and to appeal for urgent policy actions to enhance health and social protection for these urban, unprivileged socio-economic groups.

## 2. Materials and Methods

### 2.1. The Study Area

We selected Bangkok city as the study area for this research. Bangkok is the most polluted city in Thailand where air pollution is caused by diesel fumes from the city’s suffocating traffic, agricultural burning when farmers clear agricultural waste before the next harvest, and construction and manufacturing. Bangkok is represented by both formal and informal economic sectors. Informal employment represents around 28% of employment in Bangkok; most informal employment and domestic workers are home-based workers, motorcycle taxi drivers, market traders, and street vendors. More women are involved in the informal sector than men, and 65–100% of women in different job categories work more than 40 h a week [9]. Street vendors are the most likely occupation to report work-related injuries in Bangkok (20%). They are likely exposed to daily black carbon spewed by diesel engines of city vehicles, such as cars, trucks, and boats. Bangkok has found that PM_2.5_ in the air often escalated to unsafe levels—one of the leading environmental causes of premature deaths.

The Thon Buri, Din Daeng, Bang Khun Thain, Klong Toei, and Nong Chok districts in Bangkok city of Thailand were selected as the study area. The five districts represent diverse socio-economic activities and socio-cultural and economic groups. Thon Buri is the area concentrated with tourist activities due to its historical sites and cultural areas. Klong Toei has mixed economic activity of tourism, commerce, and some small industry. The Din Daeng district has dense commercial, services, and industrial zones. The three districts are in the center of Bangkok, while Bang Khun Thain and Nong Chok are peri-urban areas. The main economic activity in Bang Khun Thian is the aquaculture, fishery, and processing industry, while agricultural and rice production and the automobile industry are the main activities in Nong Chok.

**Figure 1:**
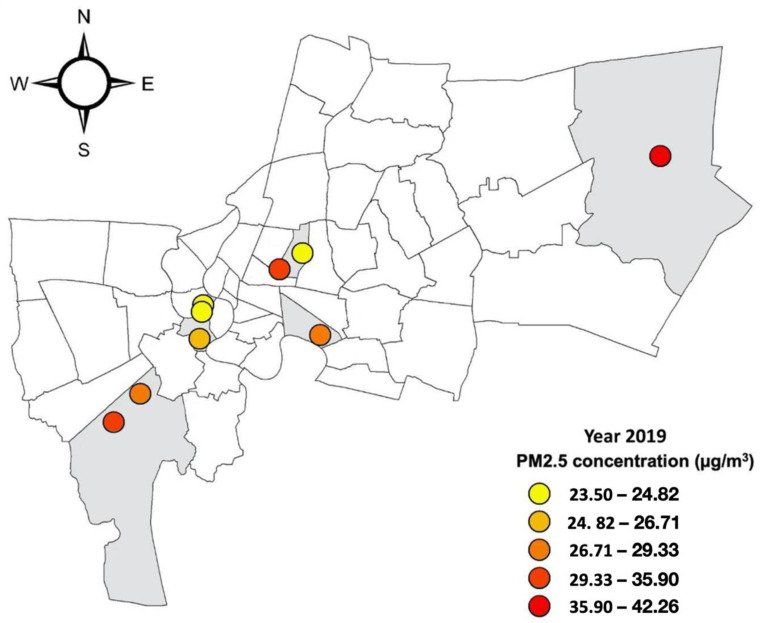
Study area and annual PM_2.5_ concentration in 2019.

According to the city’s air-monitoring data of the Thai Pollution Control Department and the Bangkok Metropolitan Administration, Din Daeng, Bang Khun Thain, and Nong Chok had 2019 annual average PM_2.5_ concentrations that measured from 35.90 to 42.26 µg/m^3,^ while Klong Toei and Thon Buri districts are less polluted areas of the city with PM_2.5_ concentrations between 23.60–24.92 µg/m^3^. The selection of two contrasting types of districts aims to explore the differences in air pollution-related health impacts and perceptions of air quality among different socio-economic groups in different geographic and polluted areas (Figure 1).

### 2.2. Data Collection 

**Figure 2:**
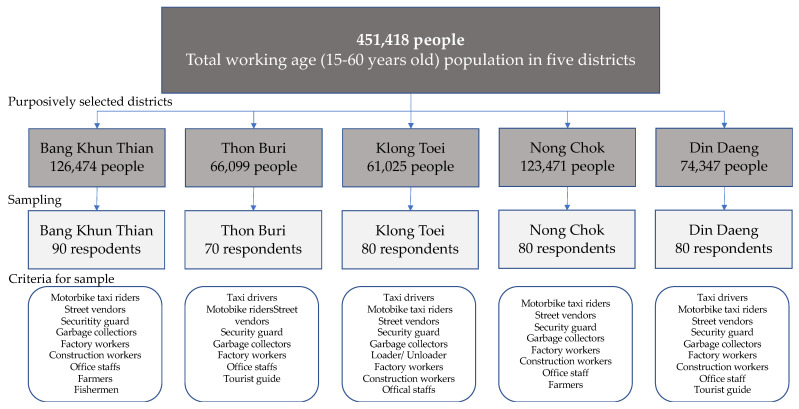
Sample procedure of the study.

Questionnaire surveys were conducted in the five districts mentioned above with 400 respondents. The total working-age population among 15 to 60 years old in five districts is 451,418 people. The sample of this research was calculated by using Yamane’s (Yamane, 1973) formula with a 95% confidence level. Thus, the sample size determined was 384 people. To increase the reliability of the data analyses, we oversampled cases to 400. To cover all socioeconomic characteristics of the worker population in each district, some other criteria of respondent selection were also set up, which included both males and females, both outdoor and indoor working environments, formal and informal sector work, all job categories, and different working locations. The sampling procedure is presented in Figure 2.

The respondent selection was made randomly by approaching them at their workplaces and asking for their voluntary participation in the survey. No payment was given to the respondents, but an N95 mask package was gifted to compensate for their time. 

The questionnaire survey consisted of three parts:

(1) Demographic information included age, gender, nationality, occupancy, education, family member, marital status, and working and living locations;

(2) Common health symptoms faced in the last years (even before the COVID-19 pandemic). Respondents were asked to write down the health symptoms and health diseases they had;

(3) Perceptions of air quality around the working environment, with ratings from toxic, unhealthy, unhealthy for sensitive people, moderate and good.

### 2.3. Data Analysis

Descriptive statistics were applied for the analysis of questionnaire survey data. The frequencies and percentages were mainly calculated to quantify respondents’ perceptions of air quality around their working environment and their health symptoms related to continuous exposure to low air quality among different socio-economic groups. Multivariate Probit regression (1) was used to estimate the degree to which the various independent variables (i.e., socio-economic and demographic factors) and various dependent variables (i.e., health symptoms) are linearly related to each other.

*y*_1_, *y*_2_,...,*y*_12_ = *f*(*x*_1_,*x*_2_,...,*x*_10_) (1)

where *y*_1_, *y*_2_,...,*y*_12_ = 12 binary dependent variables of health symptoms.

The *x*_1_,*x*_2_,...,*x*_10_ = 10 independent variables are described and coded in Table 1.

## 3. Results

### 3.1. Socio-Economic and Demographic Characteristics of the Respondents

Table 1 shows the socio-economic characteristics of the respondents. The sample represents males (57%) and females (43%), formal sector workers (42%), and informal sector workers (59%); and the even distribution among all ranges of working age groups. There were no illiterate respondents, most held secondary school degrees. An even distribution of all types of occupations and more outdoor workers than indoor workers were surveyed. Most respondents had monthly incomes of 10,000–15,000 baht, were aged 30 to 46, and worked near streets and highways. Most of them lived in towns or near streets and highways.

### 3.2. Socio-Economic Groups’ Perceptions of Air Quality in Bangkok

**Figure 3:**
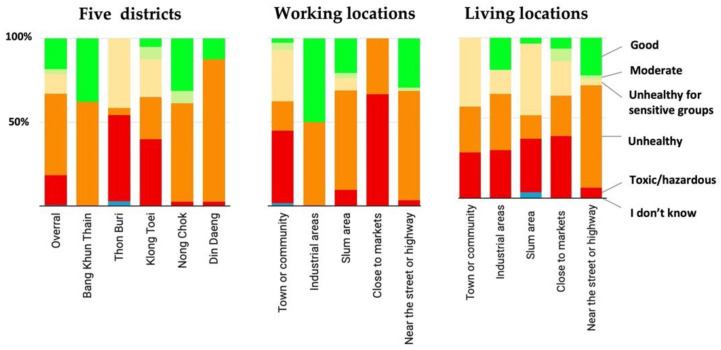
Socio-economic groups’ perceptions of urban air quality (N = 400).

In general, a very high percentage of respondents (more than 60%) considered the air quality around their working place toxic and unhealthy. Within that percentage, more than 50% of respondents in Thon Buri and around 40% of respondents in Klong Toei rated the air quality in these places as toxic. Bang Khun Thian, Nong Chok, and Diang Daeng had the smallest percentages and considered the air quality in their places toxic; almost 85% of respondents in Ding Daeng, 60% in Bang Khun Thian, and more than 50% in Nong Chok rated the air quality in their districts unhealthy. No respondents in Thon Buri considered their places to have good air quality, while nearly 40% of respondents in Bang Khun Thian and Nong Chok saw the air quality in their districts as good (Figure 3).

### 3.3. Low Air Quality-Related Health Symptoms among Urban Working Locations 

**Figure 4:**
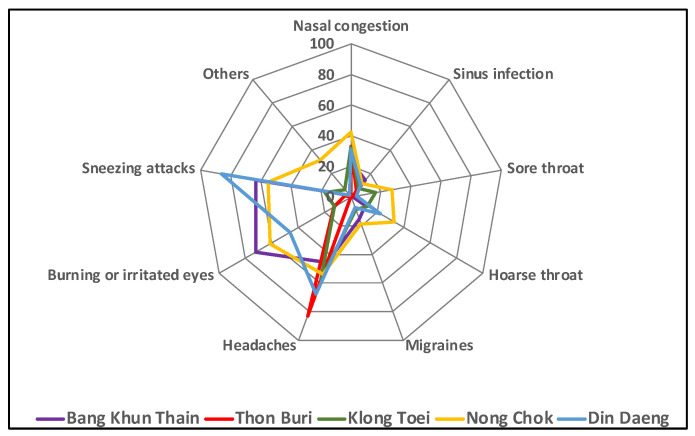
Health symptoms of socio-economic groups among five districts.

Figure 4 shows common health symptoms linked to low air quality encountered by the respondents. The surveyed data showed that common health symptoms caused by low air quality confided by respondents are headaches (61%), sneezing attacks (47%), burning or irritated eyes (43%), nasal congestion (32), hoarse throat (16%), migraines (11%) sinus infections (10%), and sore throat (10%). Bang Khun Thian, Nong Chok, and Ding Daeng are the three districts that have more respondents facing one or more of above low air quality-related health symptoms. Fewer health symptoms related to low air quality were found in Thon Buri and Klong Toei districts; however, almost 80% of respondents in Thon Buri declared they often had headaches. The survey results also revealed that one respondent in Nong Chok and five in Din Daeng indicated they had asthma, and two in Bang Khun Thian, four in Nong Chok, and two in Din Daeng had allergic rhinitis. In contrast, no respondents in the two other districts declared having those two mentioned diseases. 

**Figure 5:**
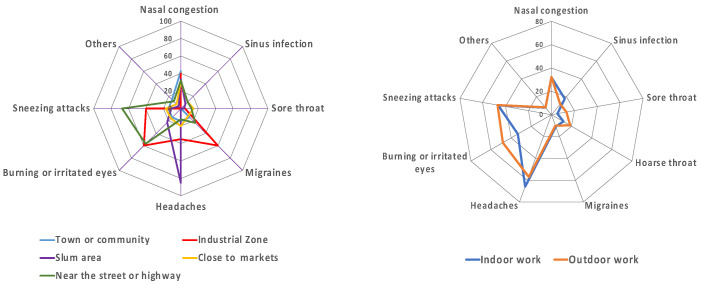
Health symptoms of socio-economic groups among different work environments.

Figure 5 depicts the different frequencies in low air quality-related health symptoms among different working places and between indoor and outdoor environments. A high percentage of respondents working near the streets, highways, and industrial zones declared they often had sneezing attacks, burning or irritated eyes, and migraines. In contrast, 85% of respondents working in slum zones often had headaches. Both indoor and outdoor workers had similar symptoms, such as sneezing, burning and irritated eyes, and headaches, but a higher percentage of the outdoor worker group than in the indoor worker group had burning or irritated eye problems.

### 3.4. Low Air Quality-Related Health Symptoms among Different Socio-Economic Groups

**Figure 6:**
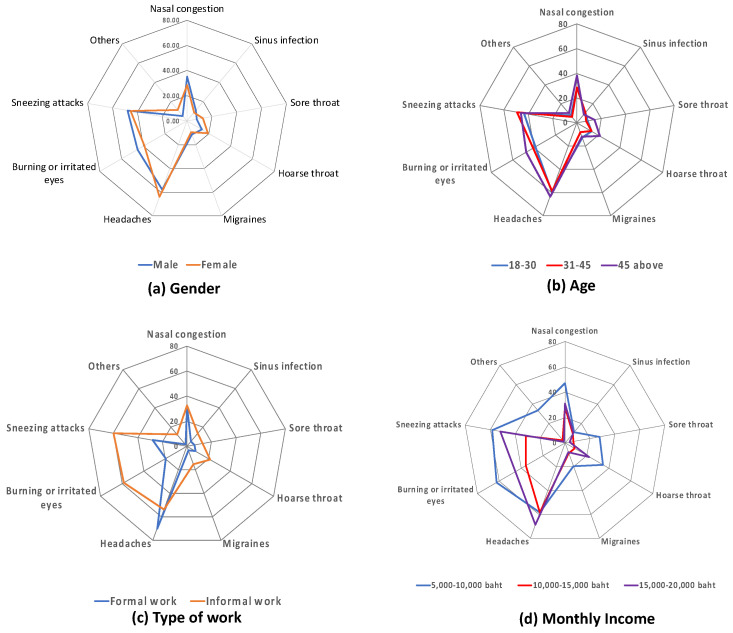
Health symptoms of different socio-economic groups.

Figure 6 depicts the similarities and differences in frequencies of health symptoms faced by different socio-economic groups. The results show no differences between the male and female groups regarding health symptoms; a similar percentage of males and females who had headaches, burning or irritated eyes, and sneezing attacks were found (Figure 6a). No differences were found among the three age groups (Figure 6b). However, a very higher percentage of informal employment respondents had one or more symptoms, such as sneezing attacks, headaches, burning or irritated eyes, hoarse throat, and nasal congestion, than the formal employment worker group (Figure 6c). The lower income groups were also found to have more health symptoms than other higher income groups (Figure 6d).

### 3.5. Factors Influencing Declared Health Symptoms among Socio-Economic Groups

Table 2 presents multivariate regression analysis results on twelve health symptoms related to low are quality variables. In general, Nong Chok is associated with many health symptoms, such as sore throat (β = 0.177, *p* = 0.033), hoarse throat (β = 0.251, *p* = 0.015), migraines (β = 0.152, *p* = 0.090), burning eyes (β = 0.340, *p* = 0.007), and sneezing attacks (β = 0.337, *p* = 0.004). Bang Khun Thian is strongly linked to burning eyes (β = 0.417, *p* = 0.001) and sneezing attacks (β = 0.438, *p* = 0.000). Din Daeng is also strongly associated with sneezing attacks (β = 0.681, *p* = 0.000) and hoarse throat (β = 0.186, *p* = 0.050). Formal sector workers are negatively associated with many health symptoms. Significant relationships are found between working location in industrial zones and burning and irritated eyes (β = 0.264, *p* = 0.045) and sneezing attacks (β = 0.292, *p* = 0.017). Working close to streets and highways is also associated with sneezing attacks (β = 0.236, *p* = 0.008). Older people are more likely to be associated with nasal congestion (β = 0.006, *p* = 0.013). In terms of job types, factory/construction worker is associated with many health symptoms; and factory/construction worker (β = 0.0.186, *p* = 0.020), farmer (β = 0.289, *p* = 0.015), and service provider (β = 0.204, *p* = 0.013) are more associated with burning/ irritated eyes.

## 4. Discussion

### 4.1. Symmetry and Asymmetry between Air Quality-Monitoring Data and Different Socio-Economic Groups’ Health Symptoms and Perception of Air Quality 

The findings showed the symmetry between air quality-monitoring data and common low air quality-related health symptoms encountered by the respondents. The three districts, namely Ding Daeng, Nong Chok, and Bang Khun Thian, with alarming PM_2.5_ concentrations from 35.90 to 42.26 µg/m^3^, have more respondents with one or more health symptoms. Klong Toei and Thon Buri districts are less polluted areas in the city with PM_2.5_ concentrations between 23.60–24.92 µg/m^3^, evidently having fewer people with low air quality-related health symptoms. This confirmed the impact of air pollution on human health and well-being demonstrated by much research in many countries [6], which showed air pollution caused chronic diseases, such as chronic asthma, pulmonary insufficiency, cardiovascular diseases, cancer and cardiovascular both in the short term and long term. Nitrogen oxide, sulfur dioxide, volatile organic compounds, carbon monoxide, heavy metals, and polycyclic aromatic hydrocarbons from polluted air emissions are all considered air pollutants harmful to humans [17].

However, the findings showed the asymmetry between the respondents’ perceptions of air quality around their environment and the air quality-monitoring data. Although Bang Khun Thian, Nong Chok, and Diang Daeng are the most polluted districts with very high PM_2.5_ concentrations, very few numbers in these three districts have considered the air quality in their districts to be toxic. On the contrary, the Klong Toei and Thon Buri districts are less polluted areas, but around 40–50% of respondents in Thon Buri and Klong Toei rated the air quality in these places as toxic. No respondents in Thon Buri thought their district air quality was good, but nearly 40% of respondents in Bang Khun Thian and Nong Chok saw the air quality in their districts as good. This finding demonstrated that the surveyed people had no information and awareness about air quality in their areas, and their perceptions of air quality were based on their observations of their environments. Because Bang Khun Thian is in the peri-urban areas where there are more open and large spaces of rice fields and aquacultural areas and Ding Daeng is found near highways, many people may think they have good air quality. Similarly, many people in Thon Buri and Klong Toei, central historical and commercial areas, the air quality might feel “unbreathable.” Many studies, e.g., [18,19], have found that air pollution affects labor market participation; however, most respondents in the most polluted district had low awareness of air pollution, and many respondents in this study were low daily wage workers. This constrains their control over the hours they choose to work, although a large number of respondents in this study are from the informal sector. Indeed, air pollution is an invisible killer [20] that cannot be easily observed by the human senses. However, air quality communication is lacking in many countries [21], and the public has little knowledge about sources of air pollution [22]. Knowledge affects practices; thus, improving public information and awareness of air pollution and its impacts on human health can motivate individuals to engage in behaviors to mitigate the health risks of air pollution [23], and communication can help to build skills that increase self-efficacy [24].

### 4.2. Inequalities of Low Air Quality-Related Health Impacts among Socioeconomic Groups in the World of Work

The research findings showed inequalities of low air quality-related health impacts on socio-economic groups in the world of work. People working near the streets, highways, and industrial zones tended to have more health symptoms related to low air quality than in other working environments. Environmental pollution is always a problem in the industrial zones because of hazardous industrial waste and emission, which creates a negative impact on human health [25], while air pollution-related high traffic in urban areas induces a threat to the population living and working near streets and highways [26]. The results also demonstrated no differences in health symptoms between indoor and outdoor work. This is true because indoor air quality is highly affected by outdoor air quality [27]. The outdoor worker group had slightly more burning and irritated eye problems than the indoor worker group because of direct contact with dust.

A much higher percentage of informal employment respondents was found to have one or more symptoms, such as sneezing attacks, headaches, burning or irritated eyes, hoarse throat, and nasal congestion than the formal employment worker group. Furthermore, the lower income groups were also found to have more health symptoms than other higher income groups. This confirmed that socioeconomic deprivation could increase diseases and mortality caused by air pollution [28], and air pollution has a greater effect on the poor population [29]. In the world of work, the informal sector workers often work in unsafe and unprotected environments with low-paid salaries and zero benefits. They have no chance to access or be trained in health protection measures or be equipped with labor safety protection toolkits. They do not have healthcare and insurance to be monitored and treated for their health problems over time.

## 5. Conclusions

This research confirms the relationship between air pollution indexes and public health. However, the awareness of air pollution and its invisible violence on human health is little known by the public. The study appeals for effective air pollution communication from governments, non-governmental organizations, and mass media to enhance the public literacy of air pollution, the sources of air pollution, and its critical health impacts. This information can help motivate the public to change behaviors toward mitigating the health risks of air pollution, and communication can help to build skills that increase self-efficacy. In addition, the findings depict a picture of inequalities of air pollution impacts in the world of work, especially in the informal sector. The informal sector workers tend to have more health risks from air pollution because they are unprotected and lack knowledge of self-protection against low air quality. Since air pollution is an invisible killer that can cause a high mortality rate for any working-age population, there is a need to provide education and information about health risks to encourage low-paid and informal sector workers to self-protect to mitigate health risks caused by air pollution. In addition, critically enhancing the abilities of primary care organizations and community health care centers to address work-related health needs are essential. Primary care should be available everywhere with sufficient capacity to reach the informal sector worker population.

## Figures and Tables

**Table 1:** Socio-economic and demographic characteristics of surveyed respondents (N = 400).

Categories (Codes) *	Respondent Number	Respondents %
**Gender**		
Female (0)	228	57.00%
Male (1)	172	43.00%
**Working age groups**		
15–30 (0)	103	25.80%
31–45 (1)	171	42.80%
Over 45 (2)	126	31.50%
**Education**		
Illiterate (0)	0	0.00%
Primary school (1)	31	34.40%
Secondary school (2)	45	50.00%
High school (3)	12	13.30%
Bachelor level (4)	2	2.20%
**Jobs**		
Office worker (0)	66	16.50%
Street vendors (1)	112	28.00%
Factory and construction worker (2)	86	21.50%
Farmer/fishermen (3)	61	15.30%
Service provider (4)	75	18.80%
**Work environment**		
Outdoor (0)	153	38.30%
Indoor (1)	247	61.80%
**Types of employment**		
Informal (0)	164	41.00%
Formal (1)	236	59.00%
**Income (bath)**		
5000–10,000 baht	72	18.00%
10,000–15,000 baht	251	62.75%
15,000–20,000 baht	77	19.25%
**Working locations**		
Town or community (0)	7	1.75%
Industrial area (1)	20	5.00%
Slum area (2)	27	6.75%
Close to markets (3)	75	18.75%
Near the street or highway (4)	227	56.75%
**Working locations**		
Town or community (0)	115	28.75%
Industrial area (1)	10	2.50%
Slum area (2)	143	35.75%
Close to markets (3)	12	3.00%
Near the street or highway (4)	120	30.00%

* Independent variable description and coding.

**Table 2:** Multivariate regression results.

	Chronic Cough	Wheezing (Except Colds)	Multiple Colds	Shortness of Breath	Nasal Congestion	Sinus	Sore Throat	Hoarse Throat	Migraine	Headaches	Burning Eyes	Sneezing Attacks
**Districts**	**β (*SE*)**	**β (*SE*)**	**β (*SE*)**	**β (*SE*)**	**β (*SE*)**	**β (*SE*)**	**β (*SE*)**	**β (*SE*)**	**β (*SE*)**	**β (*SE*)**	**β (*SE*)**	**β (*SE*)**
Thon Buri ^a^												
Bang Khun Thain	−0.022 (0.04)	−0.017 (0.04)	−0.029 (0.07)	−0.089 (0.07)	−0.308 (0.14) *	−0.078 (0.09)	−0.056 (0.08)	0.042 (0.11)	0.066 (0.09)	−0.497 (0.14) **	0.417 (0.13) ***	0.438 (0.12) ***
Klong Toei	0.017 (0.03)	0.023 (0.03)	0.077 (0.05)	0.047 (0.04)	0.080 (0.09)	−0.009 (0.06)	0.139 (0.06) *	0.122 (0.070	0.126 (0.06) *	−0.199 (0.10) *	0..014 (0.09)	0.120 (0.08)
Nong Chok	0.079 (0.04)	0.026 (0.04)	0.243 (0.07) ***	0.100 (0.07)	−0.028 (0.14)	−0.073 (0.09)	0.177 (0.8) *	0.251 (0.10) *	0.152 (0.09)	−0.346 (0.14) *	0.340 (0.13) ***	0.337 (0.12) **
Din Daeng	0.000 (0.09)	0.021 (0.08)	0.016 (0.06)	0.004 (0.06)	−0.084 (0.12)	−0.007 (0.08)	−0.039 (0.08)	0.186 (0.09) *	0.079 (0.08)	−0.150 (0.13)	0.214 (0.03)	0.681 (0.11) ***
**Working location**												
Town and community ^a^												
Industrial zone	0.002 (0.04)	0.000 (0.04)	−0.061 (0.07)	−0.022 (0.07)	0.066 (0.14)	−0.026 (0.09)	0.086 (0.09)	−0.029 (0.11)	−0.135 (0.09)	−0.184 (0.15)	0.264 (0.13) *	0.292 (0.12) *
Slum areas	0.013 (0.04)	0.003 (0.04)	0.040 (0.06)	−0.002 (0.06)	0.055 (012)	0.031 (0.08)	00.080 (0.08)	0.056 (0.09)	−0.117 (0.08)	0.050 (0.13)	0.091 (0.12)	0.113 (0.11)
Close to market	0.034 (0.04)	0.042 (0.03)	0.053 (0.05)	0.036 (0.05)	0.003 (0.11)	0.055 (0.07)	0.080 (0.07)	0.059 (0.09)	−0.134 (0.07)	−0.033 (0.12)	0.002 (0.10)	0.143 (0.10)
Close to street/highways	0.020 (0.03)	0.000 (0.03)	−0.026 (0.05)	0.012 (0.05)	−0.016 (0.10)	0.050 (0.07)	0.105 (0.06)	0.082 (0.08)	−0.077 (0.07)	−0.120 (0.110	0.117 (0.100)	0.236 (0.09) **
**Living location**												
Town and community ^a^												
Industrial zone	−0.060 (0.11)	0–0.023 (0.10)	−0.138 (0.017)	−0.059 (0.17)	−0.308 (0.36)	−0.010 (0.23)	−0.109 (0.22)	−0.236 (0.27)	−0.064 (0.24)	−0.062 (0.37)	0.062 (0.33)	−0.148 (0.31)
Slum areas	−0.02 (0.03))	−0.022 (0.03)	0.051 (0.05)	−0.015 (0.05)	0.129 (0.10)	−0.042 (0.07)	0.033 (0.06)	−0.002 (0.08)	−0.015 (0.07)	0.115 (0.11)	0.047 (0.10)	−0.040 (0.09)
Close to market	−0.018 (0.09)	(−0.011 (0.08)	−0.011 (0.013)	−0.007 (0.13)	0.398 (0.28)	−0.087 (0.18)	0.028 (0.17)	−0.016 (0.21)	−0.109 (0.18)	−0.345 (0.29)	0.011 ((0.26)	0.119 (0.24)
Close to street/highways	−0.041 (0.04)	0.007 (0.03)	−0.036 (0.06)	0.004 (0.06)	−0.009 (0.12)	−0.002 (0.08)	0.047 (0.07)	−0.042 (0.09)	−0.028 (0.08)	0.040 (0.12)	0.021 (0.11)	−0.094 (0.10)
**Age**	0.001 (0.00)	0.000 (0.00)	0.000 (0.00)	−0.001 (0.00)	0.006 (0.00) *	−0.002 (0.00)	0.001 (0.00)	0.003 (0.00)	−0.003 (0.00)	0.003 (0.00)	0.000 (0.00)	0.003 (0.00)
**Gender**												
Female ^a^												
Male	0.004 (0.02)	0.000 (0.01)	−0.040 (0.03)	−0.030 (0.03)	0.100 (0.05)	0.003 (0.03)	−0.046 (0.03)	−0.067 (0.04)	0.007 (0.03)	−0.010 (0.05)	−0.017 (0.5)	−0.006 (0.4)
**Work environment**												
Informal job ^a^												
Indoor job	0.021 (0.02)	0.033 (0.02)	0.05 (0.03))	0.018 (0.03)	0.140 (0.07) *	−0.021 (0.05)	0.010 (0.04)	−0.001 (0.05)	0.044 (0.05)	0.092 (0.07)	−0.114 (0.07)	0.030 (0.06)
**Types of employment sectors**												
Informal sector work ^a^												
Formal sector work	−0.056 (0.03) *	−0.045 (0.02) *	−0.101 (0.04) *	−0.113 (0.04) ***	−0.223 (0.08) ***	−0.146 (0.05) **	−0.021 90.05)	−0.111 (0.06)	−0.072 (0.05)	−0.126 (0.08)	−0.109 (0.07)	−0.217 (0.07) **
**Income**	0.000 (0.02)	0.000 (0.00)	0.000 (0.00)	0.000 (0.00)	0.000 (0.00)	0.000 (0.00)	0.000 (0.00)	0.000 (0.00)	0.000 (0.00)	0.000 (0.00)	0.000 (0.00)	0.000 (0.00)
**Education**	0.009 (0.01)	−0.015 (0.01)	0.010 (0.02)	−0.020 (0.02)	0.046 (0.03)	−0.014 (0.02)	−0.008 90.02)	−0.003 (0.03)	−0.020 (0.020	0.016 (0.04)	0.033 (0.03)	0.044 (0.03)
**Job types**												
Office worker ^a^												
Street seller	0.012 (0.02)	−0.014 (.02)	0.013 (0.04)	0.022 (0.04)	0.002 (0.08)	0.107 (0.05) *	0.002 (0.05)	−0.057 (0.06)	−0.001 (0.05)	−0.069 (0.08)	0.109 (0.7)	−0.097 (0.07)
Factory/ Construction worker	0.062 (0.03) *	0.018 (0.02)	0.085 (0.04) *	0.077 (0.04)	0.023 (0.09)	0.126 (0.06) *	0.101 (0.05)	0.037 (0.07)	−0.007 (0.06)	−0.200 (0.09) *	0.186 (0.8) *	0.016 (0.07)
Farmer	0.02 (0.04))	0.008 (0.04)	0.006 (0.06)	0.083 (0.06)	0.050 (0.13)	0.162 (0.08) *	0.138 (0.08)	−0.042 (0.10)	0.102 (0.08)	−0.079 (0.13)	0.289 (0.12) *	−0.131 (0.11)
Service provider	0.020 (0.03)	0.054 (0.02) *	0.068 (0.04)	0.055 (0.04)	0.123 (0.09)	0.034 (0.06)	0.182 (0.08) ***	0.075 (0.07)	−0.000 (0.06)	−0.096 (0.09)	0.204 (0.8) *	0.019 (0.08)
_cons	−0.019 (0.09)	0.008 (0.08)	0.032 (0.13)	0.075 (0.013)	0.130 (0.27)	0.167 (0.17)	−0.152 (0.16)	0.008 (0.20)	0.217 (0.18)	0.786 (0.28) *	−0.188 (0.25)	0.128 (0.23)
N	400	400	400	400	400	400	400	400	400	400	400	400
Parms	23	23	23	23	23	23	23	23	23	23	23	23
RMSE	0.146	0.130	0.223	0.220	0.459	0.298	0.279	0.350	0.303	0.475	0.427	0.395
R-sq	0.090	0.071	0.200	0.121	0.093	0.068	0.182	0.142	0.079	0.110	0.296	0.411
F	1.689	1.318	4.277	2.359	1.760	1.250	3.813	2.837	1.463	2.128	7.212	11.933
*p*-value	0.028*	0.155	0.000 ***	0.001 ***	0.019 *	0.202	0.000 ***	0.000 ***	0.083	0.003 ***	0.000	0.000 ***

^a^ Reference groups. * Significant level of 0.05 (*p* ≤ 0.05), ** significant level of 0.01 (*p* ≤ 0.01), *** significant level of 0.001, (*p* ≤ 0.001).

## Data Availability

Not applicable.
